# Cultural adaptation of the Retirement Resources Inventory for Brazilian culture

**DOI:** 10.11606/s1518-8787.2019053000863

**Published:** 2019-07-10

**Authors:** Raquel Gvozd, Mariana Angela Rossaneis, Paloma de Souza Cavalcante Pissinati, Edinêis de Brito Guirardello, Maria do Carmo Fernandez Lourenço Haddad

**Affiliations:** I Universidade Estadual de Londrina. Departamento de Enfermagem. Londrina, PR, Brasil; II Prefeitura Municipal de Rolândia. Secretaria Municipal de Saúde. Rolândia, PR, Brasil; III Universidade Estadual de Campinas. Faculdade de Enfermagem. Campinas, SP, Brasil; IV Universidade Estadual de Londrina. Departamento de Enfermagem. Londrina, PR, Brasil

**Keywords:** Retirement, Aging, Surveys and Questionnaires, Translations, Validation Studies, Occupational Health, Aposentadoria, Envelhecimento, Traduções, Inquéritos e Questionários, Estudos de Validação, Saúde do Trabalhador

## Abstract

**OBJECTIVE:**

To translate and adapt the Retirement Resources Inventory for Brazilian culture.

**METHODS:**

Methodological research including the stages of translation, synthesis, evaluation by committee of judges, back-translation and pre-test. The internal consistency of the instrument with Cronbach’s alpha coefficient was evaluated.

**RESULTS:**

We considered the stages of translation and cultural adaptation adequate. The evaluation of the synthesis version by the judges resulted in the need to change 95.0% of the items to ensure the semantic, idiomatic, cultural and conceptual equivalence between the original and translated versions. In general consensus of the instrument, the agreement rate among the judges for the equivalences was 84.4%. As for the pre-test stage, 25 pre-retirees participated. The participants suggested adjustments in the instrument. The instrument’s internal consistency was 0.85. The mean time to fill in the instrument was 18.7 minutes

**CONCLUSIONS:**

The methodological process of cultural adaptation of the Retirement Resources Inventory resulted in adequate content validity and ease of understanding by the participants. We emphasize that this study precedes the evaluation process of the psychometric properties of the instrument, which will be carried out in new studies.

## RESUMO

**OBJETIVO:**

Traduzir e adaptar o *Retirement Resources Inventory* para a cultura brasileira.

**MÉTODOS:**

Pesquisa metodológica incluindo as etapas de tradução, síntese, avaliação por comitê de juízes, retrotradução e pré-teste. A consistência interna do instrumento foi avaliada pelo coeficiente alfa de Cronbach.

**RESULTADOS:**

As etapas de tradução e adaptação cultural foram consideradas adequadas, e a avaliação da versão síntese pelos juízes resultou na necessidade de alteração de 95,0% dos itens para assegurar as equivalências semântica, idiomática, cultural e conceitual entre as versões original e traduzida. No consenso geral do instrumento, a taxa de concordância entre os juízes para as equivalências foi de 84,4%. Quanto à etapa de pré-teste, participaram 25 pré-aposentados, que sugeriram adequações no instrumento. O tempo médio para preenchimento do instrumento foi de 18,7 minutos. A consistência interna global do instrumento foi de 0,85.

**CONCLUSÕES:**

O processo metodológico de adaptação cultural do *Retirement Resources Inventory* resultou em adequada validade de conteúdo e facilidade de compreensão pelos participantes. Ressalta-se que este estudo antecede o processo de avaliação das propriedades psicométricas do instrumento, que será realizado em novas pesquisas.

## INTRODUCTION

Retirement is a period of great transition in a person’s life^1^ . It can represent a positive achievement for promoting quality of life and life expectancy. However, instabilities related to social guarantees and health can arise, in addition to a negative connotation, especially due to the unproductiveness and financial dependence that some people experience when they reach this stage of life^2^ .

Uncertainties and insecurities may arise during the period that precedes retirement. On the other hand, it represents the opportunity for changes in behavior and habits that contribute to future well-being^3^ . For this trajectory to be successful, risk and protective factors that interfere with this transition must be considered^4^ . Studies analyze the preparation and adaptation to retirement by observing personal, psychosocial and organizational characteristics that affect these processes^5 , 6^ .

The literature is not unanimous about when the worker is considered pre-retired. The *Política Nacional do Idoso* (Government Policies for Older Adults)^7^ proposes the creation and maintenance of retirement preparation programs in the public and private sectors, at least two years before the work leave. Likewise, the *Estatuto do Idoso* (Older Adults Statute) offers stimulatory programs to pre-retired workers to carry out new projects at least one year before retirement^8^ .

Population ageing, with the increasing inversion of the age pyramid, has indicated a greater worker permanence in the labor market. Estimates have shown that the ageing index (AI), obtained by the ratio between the older population and the young, will increase significantly in the coming years. AI in Brazil was 44.8 and could reach 208.7 by 2050^9^ .

Given the high rate of ageing, organizations must offer retirement preparation programs (RPP) and promote healthy ageing for their employees. Researchers recommend for people who do not wish to continue working to have at least one retirement work activity, and RPPs can help them organize their time so as to incorporate other aspects relevant to their well-being into the everyday life, so they can assume certain roles when retired^10 , 11^ .

Studies on ageing and retirement are relevant not only to guide people’s management policies in public and private companies, but also to assist people in planning, deciding between retiring or continuing to work and achieving well-being in retirement^12^ . Thus, measuring and understanding how the worker perceives himself before retirement can help him prepare for situations that will be faced in the post-career period. Specific instruments for such purpose can constitute a valuable management tool.

An integrative review study, whose purpose was to identify in the literature measuring instruments that address aspects of pre-retirement or retirement published in English and Portuguese from 1976 to 2014, pointed out 28 instruments that evaluate the attitudes of planning, decision-making, adjustment, or satisfaction with retirement, four of them validated for Brazilian culture. The study made reference to an Australian instrument that can assist in the evaluation of the process that precedes retirement, since it presents an approach to healthy ageing: the Retirement Resources Inventory (RRI)^13^ . We have chosen RRI for cultural adaptation and validation because we identified the necessity for an instrument for Brazilian culture that helped with the structuring of retirement preparation strategies for healthy ageing and in promoting the worker’s quality of life.

This instrument was developed by Leung and Earl to measure the resources that should be incorporated in the planning of retirement and help with the design of appropriate interventions to identify specific deficits of this phase of life. It consists of 35 items distributed in three domains: RT1, which evaluates the emotional, cognitive and motivational resources related to retirement (items 18-35); RT2, which evaluates social resources (items 9–17); and RT3, which evaluates physical and financial resources (items 1–8)^14^ .

The measurement scale used in RRI is Likert type, which varies between one and five points for each item. The higher the score of each domain, the smaller is the deficit of resources the participant presents to the respective item. RT1 can reach a minimum score of 18 and a maximum of 90 points, RT2 can range from 9 to 45 points and RT3 can range from 8 to 40 points. The global score can range from 35 to 175 points^14^ .

This study aimed to translate and adapt the Retirement Resources Inventory, considering the lack of instruments with the same purpose in Brazil.

## METHODS

This is a methodological study of translation and cultural adaptation aiming to validate an instrument that is congruent with the original but adapted to the country’s culture^15^ . For cultural adaptation, we followed the protocol for translation of the World Health Organization, including the stages: instrument’s translation, synthesis of translations, evaluation by expert committee, back-translation and pre-test with the target audience^16 , 17^ . Before carrying out this study, we contacted one of the RRI’s authors and obtained formal authorization for this process.

The first stage of the cultural adaptation was the translation of RRI into Brazilian Portuguese by two native translators, independently. One translator had technical knowledge about the subject, but nothing was informed about the study’s objectives. This stage resulted in versions T1 and T2.

The T1 and T2 versions were analyzed by two other native translators that knew the study’s objectives and were pre-retired. These translators evaluated the two translations to identify discrepancies and create a synthesis. After this analysis, the main researcher and her adviser met one translator involved to solve discrepancies, which resulted in the *Versão Português Consenso 1* (VPC1).

Subsequently, VPC1 was evaluated by a committee of judges, consisting of four nurses, a physician, a physiotherapist, a dentist, and a physical educator. We selected specialists who presented at least two of the three criteria established by the researchers, namely: English proficiency, experience in retirement and worker ageing, and experience in translation and validation of research instruments.

The judges received a questionnaire via electronic platform that presented the original RRI and the VPC1. They also received specific guidelines to evaluate whether each item of the instrument met the semantic, idiomatic, cultural and conceptual equivalences^17^ . Semantic equivalence refers to word meaning; the idiomatic, to the formulation of colloquial expressions equivalent to the language of origin; the cultural, to the different terms and everyday situations between both cultures; and the conceptual, to words that have different cultural meanings^17^ .

Each judge was requested to evaluate the items and mark one option below to show agreement to each equivalence: “yes,” “no” or “partially.” In cases of disagreement, the judge was asked to suggest adjustments. At the end, it was possible to estimate an agreement percentage between the judges. We adopted a minimum agreement rate of 80%^18^ . The main researcher and her adviser reviewed all experts’ suggestions, which resulted in the *Versão Português Consenso 2* (VPC2).

The back-translation of VPC2 was carried out by two English native translators, independently. These translators did not participate in the first stage and did not know the instrument. This stage resulted in two backward versions (R1 and R2), which were compared with the original by the main researcher and her adviser. One translated version was adopted ( *Versão Inglês Final* – VIF).

The last stage of the translation process was the pre-test, conducted with 25 participants from the target audience, selected for convenience. Both electronic and printed forms were used with different participants in the pre-test to evaluate the understanding of the instrument in each form. For this purpose, 12 pre-retirees answered the instrument in the online format and 13 in the printed format.

Three data collection instruments were used in the pre-test: a sociodemographic and occupational questionnaire, the RRI *Versão Português Consenso 2* and a questionnaire to evaluate the comprehension of the items of the instrument, its answer options, item’s relevance and relation with retirement, suggestions for reformulating instrument’s items and layout. Each participant was requested to record the time used to answer to the instrument. Finally, the original instrument’s author assessed the instrument, which resulted in the version that met the adaptation’s method criteria. Instrument’s reliability was verified from internal consistency, measured by Cronbach’s alpha.

The research was approved by the research ethics committee of the institution where the study was conducted, under Opinion 1,543,255.

## RESULTS

The first stage of cultural adaptation evolved as planned. The versions T1 and T2 presented different translations for some items, solved with other two translators. We corrected the discrepancies, which resulted in VPC1. The specialists’ evaluation of VPC1 improved several items. Table 1 shows the agreement percentage of each item, according to the equivalences evaluated.

**Table 1:** Judges’ agreement percentage for each instrument’s item, according to semantic, idiomatic, cultural and conceptual equivalences. Londrina, state of Paraná, Brazil, 2017.

Instrument Items	Equivalences				Total

	Semantic	Idiomatic	Cultural	Conceptual	
				
	%	%	%	%	%
Title	100.0	100.0	100.0	87.5	96.9
Instructions for filling 1	87.5	100.0	100.0	100.0	96.9
Instructions for filling 2	100.0	100.0	100.0	100.0	100.0
Item 1	62.5	100.0	100.0	87.5	87.5
Item 2	50.0	100.0	87.5	62.5	75.0
Item 3	75.0	100.0	87.5	87.5	87.5
Item 4	62.5	50.0	87.5	75.0	68.7
Item 5	37.5	100.0	100.0	87.5	81.2
Item 6	37.5	37.5	62.5	75.0	53.1
Item 7	37.5	62.5	75.0	87.5	65.6
Item 8	25.0	87.5	75.0	50.0	59.4
Item 9	62.5	87.5	75.0	87.5	78.1
Item 10	62.5	87.5	75.0	87.5	78.1
Item 11	37.5	87.5	62.5	75.0	65.6
Item 12	75.0	75.0	100.0	87.5	84.4
Item 13	50.0	87.5	87.5	100.0	81.2
Item 14	50.0	87.5	87.5	87.5	78.1
Item 15	75.0	100.0	100.0	100.0	93.7
Item 16	62.5	87.5	100.0	100.0	87.5
Item 17	62.5	87.5	100.0	87.5	84.4
Item 18	75.0	87.5	100.0	87.5	87.5
Item 19	62.5	87.5	87.5	100.0	84.4
Item 20	62.5	75.0	100.0	62.5	75.0
Item 21	75.0	87.5	100.0	100.0	90.6
Item 22	87.5	62.5	87.5	100.0	84.4
Item 23	100.0	87.5	100.0	100.0	96.9
Item 24	100.0	100.0	100.0	100.0	100.0
Item 25	62.5	100.0	100.0	100.0	90.6
Item 26	75.0	100.0	100.0	100.0	93.7
Item 27	75.0	100.0	100.0	87.5	90.6
Item 28	75.0	100.0	100.0	100.0	93.7
Item 29	75.0	87.5	100.0	87.5	87.5
Item 30	62.5	87.5	100.0	87.5	84.4
Item 31	87.5	87.5	100.0	100.0	93.7
Item 32	87.5	87.5	87.5	100.0	90.6
Item 33	100.0	87.5	100.0	100.0	96.9
Item 34	50.0	75.0	75.0	75.0	68.7
Item 35	100.0	87.5	100.0	100.0	96.9

Total	69.1	87.2	92.1	89.5	84.4

The judges’ agreement percentage for the instrument was 84.4%. Out of 38 evaluated items, 10 (26.3%) did not reach the minimum of 80% desired for the study. Most agreement rates with values below 80% refer to semantic equivalence, present in 28 items. The other equivalences presented rates below 80% in seven items each. Items 6, 7, 8 and 34 did not reach the agreement rate adopted for any equivalences. The judges suggested adjustments in 95.0% of the items, adjustments related to the exchange of words and expressions by synonyms more applicable to the Brazilian culture, and addition, removal or substitution of words and expressions to improve sentence comprehension ( Box ).

**Box:** Description of items modified as suggested by the judges committee. Londrina, state of Paraná, Brazil, 2017.

Item	Original document	Version Português Consenso 1, resulted from the synthesis of translations	Version Português Consenso 2, resulted from judges committee and used in pre-test
Title	Instrument Retirement Resources Inventory	*Instrumento Inventário de Recursos para a Aposentadoria*	*Inventário de Recursos para a Aposentadoria*
Instructions 1	The RRI consists of resource items across 3 domains: RT3 (physical and financial), RT2 (social), and RT1 (emotional, cognitive and motivational).	*O IRA avalia recursos em três domínios: RT3 (físicos e financeiros), RT2 (sociais), RT1 (emocionais, cogni* *tivos e motivacionais).*	*O IRA avalia recursos em três domínios: RT3 (físico e financeiro), RT2 (social), RT1 (emocional, cognitivo e motivacional).*
1	… my general health condition… ( ) average	*… meu estado geral de saúde…* *( ) médio*	*… minha condição geral de saúde…* *( ) regular*
2	I am _______ affected by one or more… ( ) mildly ( ) moderately ( ) more than moderately ( ) severely	*Eu ______ de uma ou mais…* *( ) não sofro* *( ) sofro pouco* *( ) sofro moderadamente* *( ) sofro bastante* *( ) sofro gravemente*	*Considero que ____* *_ afetado por uma ou mais…* *( ) não sou* *( ) sou pouco* *( ) sou moderadamente* *( ) sou muito* *( ) sou muitíssimo*
3	I am _______ affected by… ( ) no ( ) mildly ( ) moderately ( ) more than moderately ( ) severely	*Eu ______ de…* *( ) sofro pouco* *( ) sofro moderadamente* *( ) sofro bastante* *( ) sofro gravemente*	*Considero que ______ afetado por…* *( ) não sou* *( ) sou pouco* *( ) sou moderadamente* *( ) sou muito* *( ) sou muitíssimo*
4	… daily activities or activities that I am interested in. ( ) limited/inadequate ( ) a moderate amount of	*… atividades do dia-a-dia ou atividades que me interessam.* *( ) limitada/pouca* *( ) considerável*	*…atividades diárias ou atividades que me interessam.* *( ) limitada/insuficiente* *( ) moderada quantidade de*
5	I possess _______ income to support my/my family living expenses. ( ) very little/no ( ) limited/inadequate ( ) a substantial amount of	*A renda/dinheiro que tenho para sustentar a mim e minha família é:* *( ) muito pouco/nenhum* *( ) p* *ouco/insuficiente* *( ) considerável*	*Possuo _______ renda para sustentar as minhas despesas e da minha família* *( ) muito pouca/nenhuma* *( ) limitada/insuficiente* *( ) razoável*
6	I have ______ financial support from my personal savings. ( ) a substantial amount of	*A quantidade de dinheiro que provém da minha poupança é:* *( ) considerável*	*Tenho _____ suporte financeiro que provém das minhas economias pessoais.* *( ) suficiente*
7	I have ______ financial support from my investments. ( ) a substantial amount of	*A quantidade de dinheiro que provém dos meus investimentos é:* *( ) considerável*	*Tenho _____ suporte financeiro proveniente dos meus investimentos* *( ) suficiente*
8	I have ______ financial support from my superannuation fund. ( ) limited/inadequate	*A quantidade de dinheiro que virá da minha aposentadoria é:* *( ) pouco/insuficiente*	*Tenho ____ suporte financeiro proveniente da minha aposentadoria* *( ) limitado/insuficiente*
9	I have _______ friends whom I can interact with regularly. ( ) very few/no ( ) a moderate number of ( ) a substantial number of ( ) many	*A quantidade de amigos com os quais eu tenho contato frequente é _________.* *( ) muito pouca/nenhuma* *( ) considerável* *( ) suficiente* *( ) grande*	*Tenho _____ amigos com os quais mantenho contato frequente.* *( ) muito poucos/nenhum* *( ) um número moderado de* *( ) um número substancial de* *( ) muitos*
10	( ) very few/no ( ) few ( ) a moderate number of ( ) a substantial number of ( ) many	*( ) muito pouca/nenhuma* *( ) pouca* *( ) considerável* *( ) suficiente* *( ) grande*	*( ) muito poucos/nenhum* *( ) poucos* *( ) um número moderado de* *( ) um número substancial de* *( ) muitos*
11	( ) very few/no ( ) few ( ) a moderate number of ( ) a substantial number of ( ) many	*( ) muito pouca/nenhuma* *( ) pouca* *( ) considerável* *( ) suficiente* *( ) gra* *nde*	*( ) muito poucas/nenhuma* *( ) poucas* *( ) um número moderado de* *( ) um número substancial de* *( ) muitas*
12	I would consider… ( ) moderately	*Eu acho que…* *( ) às vezes*	*Considero que…* *( ) moderadamente*
13	I would consider… ( ) moderately	*Eu acho que…* *( ) às vezes*	*Considero que...* *( ) moderadamente*
14	I would consider… ( ) moderately	*Eu acho que…* *( ) às vezes*	*Considero que…* *( ) moderadamente*
15	I _______ receive informational support from others, where informational support refers to receiving information or advice from someone on handling difficult circumstances, rectifying a situation, following through with a solution, following-up on a difficult event, and receiving constructive criticism.	*Eu _______ recebo ajuda dos outros para obter informações, ouvir conselhos de como agir em situações difíceis, para resolver um problema, para encontrar soluções, para me recuperar de um momento difícil ou para receber uma crítica construtiva.*	*Eu _______ recebo suporte de informação de outras pessoas, onde esse suporte se refere a receber informações ou conselhos de alguém a respeito de como lidar como situações difíceis, resolver uma situação, buscar uma solução, recuperar-se de um momento difícil e receber críticas construtivas.*
16	… where emotional support means someone was available to listen…	*… com quem conto para me escutar…*	*… que significa alguém para me escutar…*
17	… respite…	*… me substituir no cuidado à outra pessoa…*	*… me substituir em alguma atividade…*
18	I experience _______ positive emotions (i.e. interested, excited, strong, enthusiastic, proud, determined, alert, inspired, attentive, active). ( ) very little/no ( ) limited/inadequate ( ) a moderate amount of ( ) a substantial amount of ( ) excess	*Eu ______ vivencio emoções positivas (por exemplo, interesse, empolgação, força, entusiasmo, orgulho, determinação, i* *nspiração, sentir-me vivo, alerta, ativo).* *( ) nunca, quase nunca* *( ) poucas vezes* *( ) várias vezes* *( ) muitas vezes* *( ) em excesso*	*Vivencio ______ emoções positivas (por exemplo, interessado, emp* *olgado, forte, entusiasmado, orgulhoso, determinado, alerta, inspirado, atento, ativo).* *( ) muito poucas/nenhuma* *( ) poucas* *( ) um número moderado de* *( ) um número substancial de* *( ) muitas*
19	I have ( ) a moderate amount of ( ) a substantial amount of	*Eu tenho…* *( ) considerável* *( ) suficiente*	*Tenho…* *( ) moderada* *( ) substancial*
20	I possess… ( ) limited/inadequate ( ) a moderate amount of ( ) a substantial	*Eu tenho…* *( ) pouca* *( ) alguma* *( ) muita*	*Tenho…* *( ) limitada/inadequada* *( ) moderada* *( ) substancial*
21	( ) limited/inadequate ( ) a moderate amount of ( ) a substantial amount of	*( ) pouca* *( ) alguma* *( ) muita*	*( ) limitada/inadequada* *( ) moderada* *( ) substancial*
22	I have… ( ) strongly disagree ( ) disagree ( ) neutral ( ) agree ( ) strongly agree	*Eu tenho…* *( ) discordo plenamente* *( ) discordo* *( ) neutro* *( ) concordo* *( ) concordo plenamente*	*Tenho…* *( ) discordo totalmente* *( ) discordo em parte* *( ) não discordo nem concordo* *( ) concordo em parte* *( ) concordo totalmente*
23	I feel… ( ) strongly disagree ( ) disagree ( ) neutral ( ) agree ( ) strongly agree	*Eu sinto…* *( ) discordo plenamente* *( ) discordo* *( ) não discordo, nem concordo* *( ) concordo* *( ) concordo plenamente*	*Sinto…* *( ) discordo totalmente* *( ) discordo em parte* *( ) não discordo nem concordo* *( ) concordo em parte* *( ) concordo totalmente*
25	I have ______ ability to…	*Eu ______ consigo…*	*Eu ______ tenho habilidade de…*
26	…different words/concepts. ( ) limited/inadequate	*… diferentes palavras/ideias.* *( ) algumas vezes*	*... diferentes palavras/conceitos.* *( ) limitadas vezes*
27	I have… ( ) limited/inadequate ( ) a substantial amount of ( ) excess	*Eu tenho* *…* *( ) pouca* *( ) muita* *( ) grande*	*Tenho* *…* *(* *) limitada* *( ) substancial* *( ) total*
28	I would consider…	*Eu acho…*	*Considero…*
29	I have... ( ) very little/no ( ) limited/inadequate ( ) a moderate amount of ( ) excess	*Eu tenho…* *( ) quase nenhuma/nenhuma* *( ) pouca* *( ) alguma* *( ) grande*	*Tenho…* *( ) pouca/nenhuma* *( ) limitada/inadequada* *( ) moderada* *( ) total*
30	I have… ( ) very little/no ( ) limited/inadequate ( ) a moderate amount of ( ) excess	*Eu tenho…* *( ) quase nenhuma/nenhuma* *( ) pouca* *( ) alguma* *( ) grande*	*Tenho…* *( ) pouca/nenhuma* *( ) limitada/inadequada* *( ) moderada* *( ) total*
31	( ) strongly disagree ( ) strongly agree	*( ) discordo plenamente* *( ) concordo plenamente*	*( ) discordo totalmente* *( ) concordo totalmente*
32	… i keep fighting to reach my goals. ( ) strongly disagree ( ) strongly agree	*… eu continuo tentando alcançar meus objetivos.* *( ) discordo plenamente* *( ) concordo plenamente*	*… continuo lutando para alcançar meus objetivos.* *( ) discordo totalmente* *( ) concordo totalmente*
33	( ) strongly disagree ( ) strongly agree	*( ) discordo plenamente* *( ) concordo plenamente*	*( ) discordo totalmente* *( ) concordo totalmente*
34	When I get stuck on something, it’s hard for me to find a new approach. ( ) strongly disagree ( ) strongly agree	*Quando eu fico “emperrado(a)”/enrolado, é difícil encontrar uma saída/solução diferente.* *( ) discordo plenamente* *( ) concordo plenamente*	*Quando eu fico “emperrado(a) em algo”, tenho dificuldade para encontrar uma outra alternativa para prosseguir.* *( ) discordo totalmente* *( ) concordo totalmente*
35	I create… ( ) strongly disagree ( ) strongly agree	*Eu crio…* *( ) discordo plenamente* *( ) concordo plenamente*	*Crio…* *( ) discordo totalmente* *( ) concordo totalmente*

We accepted and applied all judge’s suggestions in the pre-test with the target audience. From the 25 participants, 25.0% were pre-retiree teachers and 75.0% administrative or operational technicians, predominantly women (76.6%) with an average age of 57.

At this stage, the judges suggested adjustments in statements of items 6, 7 and 8. Six participants suggested adaptations for item 6: *“Tenho _____ suporte financeiro que provém das minhas economias pessoais”* [“I have _____ financial support that comes from my personal savings”]. The item was changed to *“Tenho _____ suporte financeiro que provém das minhas economias pessoais (exemplo: poupança)”* [“I have _____ financial support that comes from my personal savings (e.g. saving account)”].

Six participants suggested adaptations in item 7: “ *Tenho_____ suporte financeiro proveniente dos meus investimentos”* [“I have_____ financial support from my investments”]. They suggested the addition of terms to exemplify the investment types, creating the new question: *“Tenho_____ suporte financeiro proveniente dos meus investimentos (exemplo: imóveis, negócios, aplicações)”* [“I have financial support from my investments (example: property, own business, applications)”].

The Item 8 ( *“Tenho_____ suporte financeiro proveniente da minha aposentadoria”* ) [“I have_____ financial support from my retirement income”] raised questions by six participants for referring specifically to retirees, and was adapted for *“O suporte financeiro que terei da minha aposentadoria será:”* [“The financial support I will have with my retirement will be:”].

Eight participants had difficulty choosing between the answer options of 22 items, which showed similarity between them and induced double answers. In addition, nine participants suggested changing the Likert alternatives of 13 questions (4, 5, 6, 7, 8, 9, 10, 11, 19, 20, 21, 29 and 30) so that only one word remained for each answer, according to example: from *“muito poucos/nenhum”* [“very few / none”] to only *“nenhum”* . RRI’s author accepted and approved these alterations.

One participant indicated changes in the statement of item 31, from *“diante das dificuldades”* [“in face of difficulties”] to *“diante de qualquer dificuldade”* [“in face of any difficulty”]. Four participants suggested replacing the term *“substancial”* [“substantial”], present in eight alternatives (9, 10, 11, 18, 19, 20, 21 and 27), by the term *“muita”* [“a lot”], because it is more appropriate for Brazilian culture. Another suggestion was the inclusion of the term *“parcialmente”* [“partially”] in the questions with Likert *“discordo”* [“disagree”] and *“concordo”* [“agree”]. All suggestions were adhered to.

Participants did not suggest changing the instrument’s layout. The mean filling time was 18.7 minutes.

The instrument’s internal consistency was 0.85 after adjustments. For the domains, the internal consistency was 0.77 for RT1, 0.70 for RT2 and 0.60 for RT3. In case of exclusion of an item, there would be no major alterations in alpha.

One of the RRI’s authors approved the Brazilian final version. The RRI version for Brazilian culture received the name *Inventário de Recursos para a Aposentadoria* (IRA – Retirement Resources Inventory).

## DISCUSSION

The methodological process of translation and cultural adaptation of RRI was successful and improved its use in Brazil in all stages adopted. The importance of rigor in the adaptation and validation of measurement instruments is emphasized, especially when dealing with different cultures^19^ .

The translation stage showed significant discrepancies, which indicated the need to consult two other translators for VPC1 to be implemented. The translators who corrected the discrepancies were in the pre-retirement phase. The authors considered it a facilitating factor in choosing the most appropriate terms, once they were familiar with the subject^16^ .

Judges’ evaluations were essential to improve the translated version according to the Brazilian culture. They indicated changes in 95.0% of the items, changes related to semantic, idiomatic, cultural and conceptual equivalence. We accepted all the suggestions because we understood that this process of content evaluation would be essential for the next stages in the process of cultural adaptation. After its conclusion, we considered that the changes refined the instrument and provided more clarity and objectivity.

We emphasize that the foreign instrument was developed and applied in a retired public. However, the authors of the original study pointed out that it would serve as an important measure of resources necessary to be implanted in pre-retirement strategies^14^ . Thus, we opted for validation in pre-retirees. This option did not bring losses to the process of cultural adaptation, since only item 8 was specific for retired individuals. Other items were also pertinent for the pre-retired public. During the pre-test, we verified the need to adapt the statement of item 8 to the pre-retired public, which was done without major problems. Changes made in this stage resulted in a greater understanding of the instrument by the target audience, making possible its future use^20^ .

Still in the pre-test, several participants indicated difficulties in differentiating options from the measurement scales, especially when the options presented more than one term or similar terms for the same answer . We made adjustments on the indicated questions, aiming at providing a better understanding of the Likert scale. A measuring instrument containing a well-defined Likert scale allows better understanding and interpretation of the results^21^ .

We were especially careful when choosing pre-retirees from different professional categories to evaluate the instrument in pre-test in order to identify its accessibility to all educational levels. The assessment also came in printed and electronic forms, which demonstrates the easy use of IRA in both forms. It is recommended in the literature online data collection to be widely used in research because it provides faster data collection, economy and good use of answers^22^ . However, it is important to emphasize that this strategy of data collection may be accompanied by a low answer rate.

As limitation we indicate the realization of only one round of instrument’s evaluation by the judges committee. The judges suggested changes in most items. A re-evaluation could minimize the need for adjustments made at the pre-test stage. Yet, we chose to proceed with the pre-test after the first round because we reached the total judges’ agreement percentage higher than 80% and considered that target audience’s evaluations in pre-test would make the instrument more sensitive to pre-retirees.

Although the evaluation of its internal consistency showed a satisfactory result, the RT3 domain presented an unsatisfactory Cronbach alpha coefficient (0.60). This coefficient may be influenced by the number of items the domain in23, as observed, in which the domains with the lowest number of items (nine in RT2 and eight in RT3) had lower internal consistency. It is also necessary to re-analyze the reliability of the instrument translated and adapted to Brazil in a larger sample than the one used in the present investigation.

RRI’s adaptation to Brazilian culture brings important contributions to the structuring of retirement programs, as it can help to measure the resources that the individual needs to improve to achieve a good post-career. We call attention to the necessity to provide public policies for an effective preparation of a retirement with life quality, including strategies of physical, mental and social well-being.

The Retirement Resources Inventory is a public domain tool available for free access. It is not necessary authorization from the authors to its use for professionals interested in research or in service, as long as they cite the instrument’s reference in their productions.

## CONCLUSION

The cultural adaptation process of the Retirement Resources Inventory followed the internationally recommended protocol, obtaining satisfactory results. The adapted version was considered adequate in relation to the semantic, idiomatic, cultural and conceptual equivalences, being approved by the original instrument’s authors.

The pre-tests performed allowed to refine the instrument, which made it more accessible to the target audience and easier to understand, without losing the original instrument’s purpose. The translated and adapted instrument is reliable for use in Brazilian pre-retirees. However, additional tests need to be conducted to evaluate the psychometric properties of this version.
